# Ethanolic Extracts of Cupressaceae Species Conifers Provide Rapid Protection against Barium Chloride-Induced Cardiac Arrhythmia

**DOI:** 10.3390/ph17081003

**Published:** 2024-07-29

**Authors:** Meng-Ting Zeng, Li-Yue Huang, Xiao-Hui Zheng, Yan-Qi Fu, Ching-Feng Weng

**Affiliations:** 1 Functional Physiology Section, Department of Basic Medical Science, Xiamen Medical College, Xiamen 361023, China; 18859868050@163.com (M.-T.Z.); hly0915@163.com (L.-Y.H.); 201500010365@xmmc.edu.cn (X.-H.Z.); xmepb1@163.com (Y.-Q.F.); 2 Institute of Respiratory Disease, Department of Basic Medical Science, Xiamen Medical College, Xiamen 361023, China; 3 LEADTEK Research, Inc., New Taipei City 235603, Taiwan

**Keywords:** Cupressaceae, antiarrhythmia, cardioprotection, herbal medicine, phytochemicals

## Abstract

Sudden cardiac death (SCD) is responsible for a high percentage of cardiovascular fatalities, with ventricular arrhythmias being the most common cause. Despite numerous clinically available antiarrhythmic drugs (AADs), AADs retain some undesirable arrhythmic effects, and their inappropriate use can lead to severe adverse reactions. The exploration of new therapeutic options against arrhythmias with fewer unreceptive effects is of utmost importance. The ethanolic extracts of seven Cupressaceae species, namely, *Chamaecyparis obtusa*, *Juniperus chinensis* (L.) Ant., *Sabina chinensis* (L.) Ant. cv. Kaizuca, *Platycladus orientalis* (L.) Franco, *Juniperus sabina* L., *Fokienia hodginsii*, and *Juniperus chinensis* ‘Pyramidalis’ were investigated for their pharmacological effects on barium chloride (BaCl_2_)-induced arrhythmia using normal II lead electrocardiogram (ECG) measurements in a mouse model. According to the ECG profiles, pretreatment with *C. obtusa*, *P. orientalis*, and *J. sabina* extracts provoked dose-dependent protection against BaCl_2_-induced arrhythmia, while pretreatment with the other four species and amiodarone did not exert cardioprotective effects. The treatment effects were confirmed using a rat model. The therapeutic effects of *C. obtusa*, *P. orientalis*, and *J. sabina* extracts on the M2 and M3 receptors but not the M1 receptor were mediated by the inhibition of the M2 receptor blocker (methoctramine tetrahydrochloride), M3 antagonist (4-DAMP), or M1 receptor blocker (pirenzepine dihydrochloride). This first-line evidence illustrates that certain Cupressaceae species possess active antiarrhythmic components. The first line of key findings revealed that active components of certain Cupressaceae species have cardioprotective effects, suggesting that these innovative phytochemicals have promising potential for preventing the occurrence of cardiac arrhythmia and reducing sudden cardiac death.

## 1. Introduction

Cardiovascular diseases (CVDs) are major causes of health problems and death, representing prominent causes of morbidity and mortality worldwide and a total health and economic burden. Numerous lines of evidence suggest that CVDs include complex remodeling responses, such as hypertension, myocardial ischemia, and valve disease, which lead to poor clinical outcomes, including arrhythmia, heart failure (HF), and sudden cardiac death (SCD). These deleterious effects not only aggravate intrinsic heart disease and influence the eminence of life of patients but also induce SCD, which is life-threatening [1]. As the utmost populous country worldwide, China has ≈290 million patients with CVDs, and thus, CVDs represent the leading cause of death in the Chinese population [2]. In most cases, SCD leads to a sudden and unpredictable death due to an alteration in heart rhythm (sudden cardiac arrest). It estimates about 300,000 to 400,000 deaths annually in the United States and approximately 600,000 deaths annually in China [3,4].

Notably, the pathogenesis of arrhythmia is complicated and unpredictable. Intervention alternatives for arrhythmias include medications, radiofrequency ablation, cardiac resynchronization, implantable cardioverter-defibrillators, artificial pacemakers, and heart transplants [5,6,7,8,9]. Notwithstanding technological advances in catheter ablation therapy, antiarrhythmic drugs (AADs) are a basis for the management of cardiac arrhythmias. Experimental and translational data have demonstrated that regularly used AADs exert numerous effects on the heart, and the manifestation of these effects intensely depends on the specific experimental or clinical settings [10]. In the clinic, the treatment of arrhythmia is mainly based on Western medicine, i.e., propafenone (calcium channel blocker) and propranolol (β receptor blocker), but medical research has shown that AADs also have undesirable arrhythmic effects and that their improper usage can frequently cause more severe adverse reactions. Amiodarone [2-butyl-3-(3′,5′-diiodo-4′α-diethylaminoethoxybenzoyl)-benzofuran] (AMD), a class III antiarrhythmic drug that is an antagonism of Na^+^, Ca^2+^, and K^+^ channels, and the β-adrenergic receptors as well, is a highly efficient AAD with potentially serious side effects in humans, such as idiosyncratic hepatotoxic reactions [11] and severe pulmonary toxicity (interstitial pneumonia and lung fibrosis) [12]. Despite numerous clinical options for AADs, pharmacotherapy is still ineffectual for the majority of patients. Additionally, all antiarrhythmic agents that act via diverse ion channels retain life-threatening proarrhythmic potential. Though the long-term consumption of these drugs can release symptoms, they can lead to an inadequate prognosis and may even upsurge mortality; hence, there is an urgent necessity to discover new antiarrhythmic treatment strategies involving natural products [13,14].

New phytoconstituent drugs and their physicochemical properties can be used for the treatment of various diseases because the secondary metabolites of plants include important bioactive compounds that are selected and propagated naturally for application as remedies against various human diseases and health disorders [15]. Remarkably, *Juniperus sabina* L. and *Sonchus oleraceus* (L.) L. are broadly utilized as traditional medicinal plants in China, and their aqueous extracts have been taken as a folk medicine to treat infections, inflammatory diseases, and tumors [16]. Several studies have shown that *J. sabina* L. significantly improves hepato-protective activity, antioxidant activity, antidiabetic activity, and antitumor effects [17,18,19]. *J. chinensis* has demonstrated noteworthy improvements in anti-proliferative and antifungal activities [20,21,22]. In addition, *J. formosana Hayata* has been shown to have an inhibitory effect on the progression of kidney disease, urinary disorders, gynecological diseases, and lung cancer [23,24,25]. The fruit and leaves of junipers are usually used as tea, and minced fruits are applied to lower blood sugar contents in Anatolia [19]. *Chamaecyparis obtusa* was found to be effective in preventing bleeding, possessing antibacterial activity, and exerting antitussive effects [26]. Whether Cupressaceae plants possess antiarrhythmia activity remains to be investigated.

In this study, we attempted to evaluate the efficacy of pretreatment with Cupressaceae in a murine model of barium chloride (BaCl_2_)-induced arrhythmia. To achieve this goal, seven Cupressaceae species, including *Chamaecyparis obtusa*, *Juniperus chinensis* (L.) Ant., *Sabina chinensis* (L.) Ant. cv. Kaizuca, *Platycladus orientalis* (L.) Franco, *Juniperus sabina* L., *Fokienia hodginsii*, and *Juniperus chinensis* ‘Pyramidalis’, were collected and extracted with ethanol. The biological function (protection) of the ethanolic extracts against BaCl_2_-induced arrhythmia was examined using normal II lead electrocardiogram (ECG) measurements in mouse models.

## 2. Results

### 2.1. A Survey of the Medicinal Value of Various Conifer Plants

The literature search of electronic databases, including PubMed, MEDLINE, ScienceDirect, Google Scholar, the Cochrane Library, and Web of Science, was performed to identify articles published from inception until May 2023, illustrating that various conifer plant extracts composed of a variety of active components have great medicinal value (Supplemental Table S1 in Supplementary File). For example, *F. hodginsii*, a rich source of structurally diverse diterpenoids, has been used for the treatment of stomach aches, nausea, vomiting, and anti-proliferative activity [27,28,29,30], which are dependent on various components. One report shows that the volatile oil (100 mg/kg body weight, p.o.) of *Cupressus funebris* Endl can significantly enhance its anti-inflammatory activity [31]. The volatile oil of *J. chinensis ‘Pyramidalis’* has significant antitumor and antibacterial effects [32]. In addition to its blood circulation, diuretic activity, and antibacterial activity, *S. chinensis* L. exerts antidiabetic effects via its α-amylase inhibitory activity [33]. The compounds β-phellandrene, terpinen-4-ol, and bornyl acetate are major constituents of essential oils in *S. chinensis* and have been found to possess effective antifungal activity [22,34]. Three phenolic compounds, cosmosiin, caffeic acid, and p-coumaric acid, were firstly isolated from the leaves of *Cupressus sempervirens* L.; together with cupressuflavone, amentoflavone, rutin, quercitrin, quercetin, and myricitrin, these compounds have been found to have high antioxidant activity, and quercetin, rutin, caffeic acid, and p-coumaric acid are further used for hepatoprotective activity [35]. The previous study affords a promising complementary alternative for the proper use of a selection of essential oil combinations for use in the respiratory tract [36]. In total, 33, 37, and 37 compounds were found in the oils from the leaves of *S chinensis* (L.) Ant, *C. lusitanica* ‘Zhongshan’ Mill, and *S. chinensis* (L.) Ant. Cv. Kaizuca, respectively. Fourteen compounds were commonly identified, such as thujene, alpha-pinene, camphene, sabinene, beta-myrcene, alpha-terpinene, gamma-terpinene, alpha-terpinolene, bornyl acetate, beta-elemene, alpha-amorphene, germacrene D, delta-cadinene, and elemol. Moreover, each species contained its own particular compounds. Remarkably, the main components are sabinene (20.99%), limonene (19.78%), and bornyl acetate (11.68%) for *S. chinensis* (L.) Ant; alpha-pinene (10.39%), sabinene (11.19%), and delta-3-carene (8.88%) for *C. lusitanica* ‘Zhongshan’ Mill; and limonene (24.56%) and beta-myrcene (8.04%) for *S. chinensis* (L.) Ant. Cv. Kaizuca [37]. Because of the various associated phytochemicals with numerous medicinal properties, diverse conifer plants might represent a new source of materials with pharmacological properties involved in protection against cardiac arrhythmia that is worth investigating.

### 2.2. Screening of Various Conifer Plant Extracts in Mice

**Figure 1:**
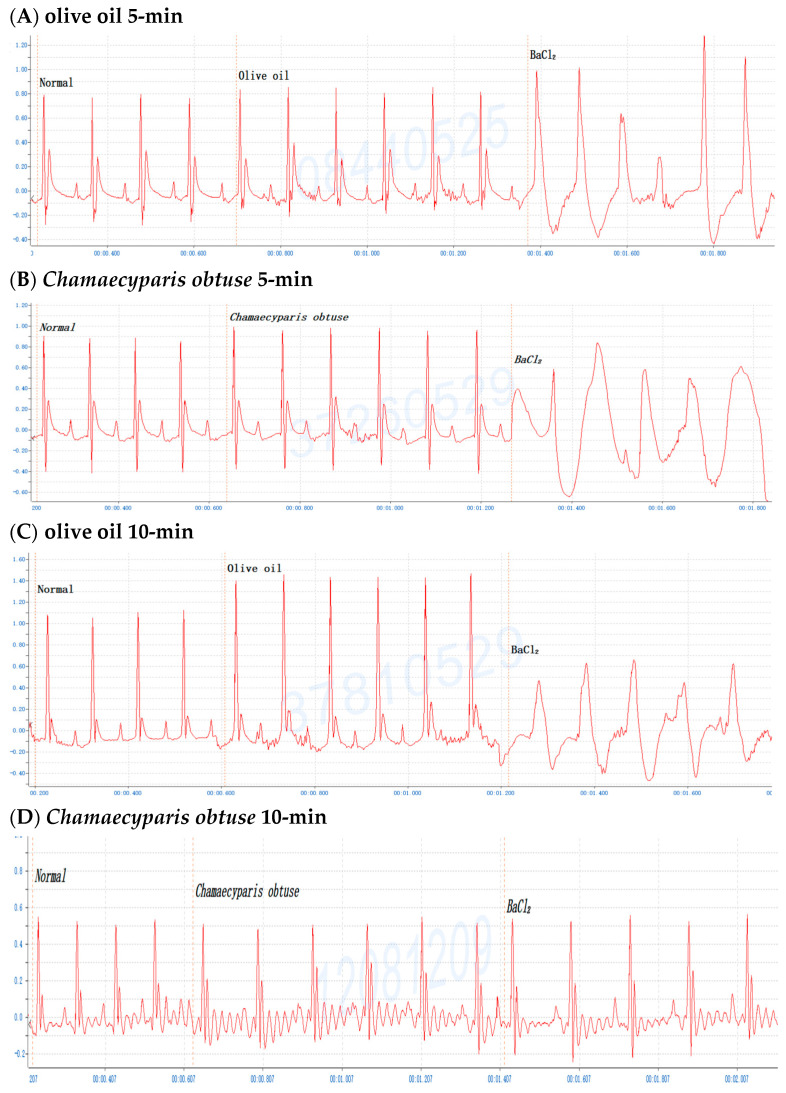

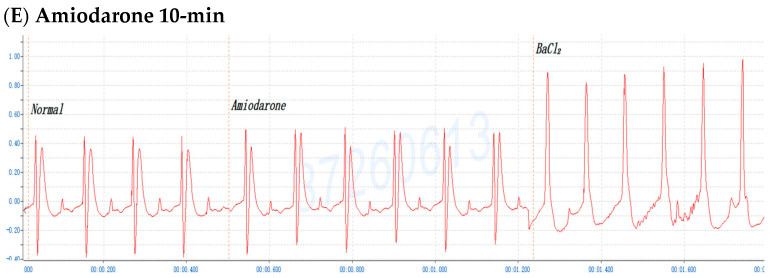
The protective effect of olive oil and *Chamaecyparis obtusa* (0.1 mL/10 g Bwt) at 5-min or 10- min after administration in mice before BaCl_2_-induced arrhythmia. (**A**) Olive oil for 5-min, (**B**) olive oil for 10-min, (**C**) *Chamaecyparis obtusa* for 5-min, (**D**) *Chamaecyparis obtusa* for 10-min, and (**E**) 0.6% amiodarone (15 mg/kg Bwt) for 10-min.

**Figure 2:**
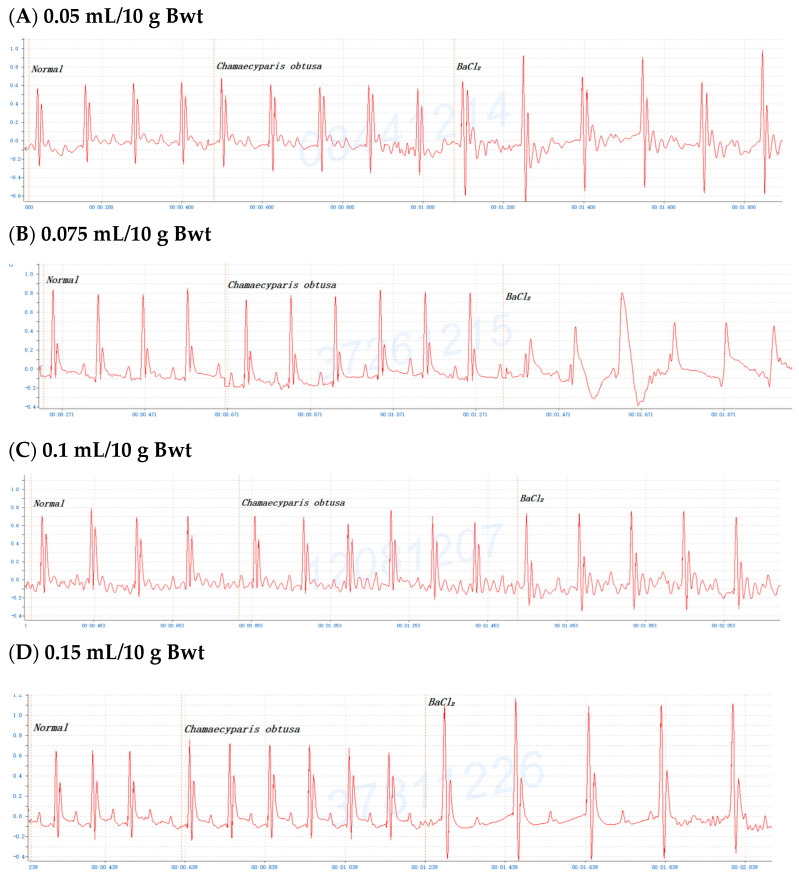

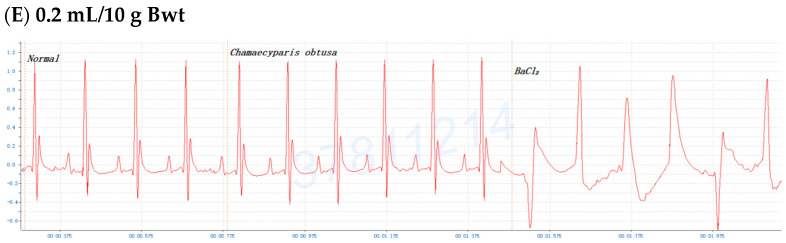
The protective effect of various concentrations of *Chamaecyparis obtusa* in mice after BaCl_2_-induced arrhythmia: (**A**) 0.05 mL/10 g Bwt, (**B**) 0.075 mL/10 g Bwt, (**C**) 0.1 mL/10 g Bwt, (**D**) 0.15 mL/10 g Bwt, and (**E**) 0.2 mL/10 g Bwt.

**Figure 3:**
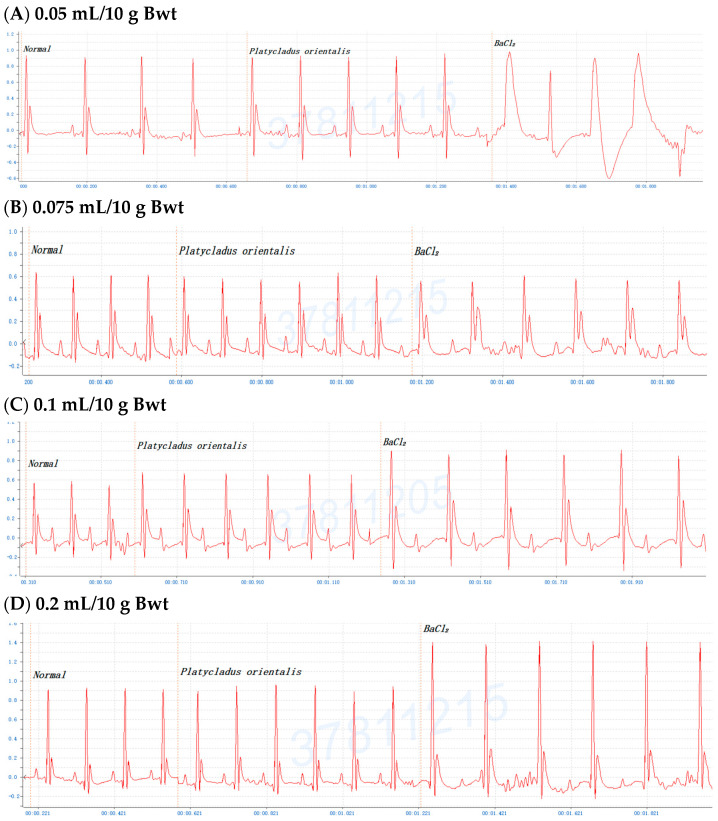
The protective effect of various concentrations of *Platycladus orientalis* in mice after BaCl_2_-induced arrhythmia: (**A**) 0.05 mL/10 g Bwt, (**B**) 0.075 mL/10 g Bwt, (**C**) 0.1 mL/10 g Bwt, and (**D**) 0.2 mL/10 g Bwt.

**Figure 4:**
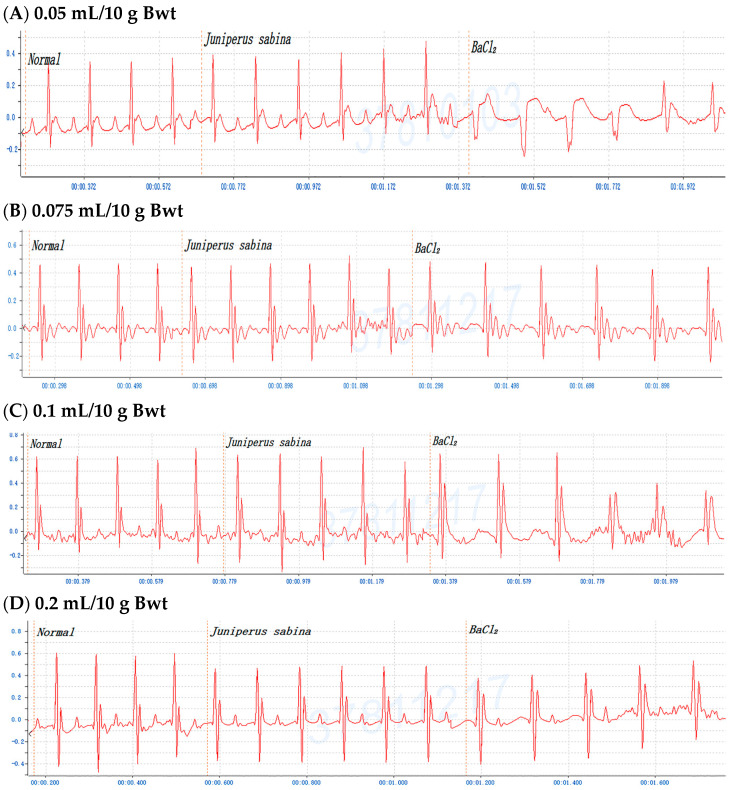
The protective effect of various concentrations of *Juniperus sabina* in mice after BaCl_2_-induced arrhythmia: (**A**) 0.05 mL/10 g Bwt, (**B**) 0.075 mL/10 g Bwt, (**C**) 0.1 mL/10 g Bwt, and (**D**) 0.2 mL/10 g Bwt.

To investigate the antiarrhythmic effects on conifer plants, mice were gavaged with the crude extracts of *F. hodginsii*, *C. obtusa*, *J. chinensis* (L.) Ant., *S. chinensis* (L.) Ant. cv. Kaizuca, *P. orientalis* (L.) Franco, *J. sabina* L., or *J. chinensis* (0.1 mL/10 g Bwt) for 10 min and injected (i.p.) with 0.8% BaCl_2_ solution (0.1 mL/10 g Bwt). First, mice were orally administered olive oil alone for 5 or 10 min before BaCl_2_ injection, and the mice with an ECG of BaCl_2_-induced arrhythmia failed to show any effect of the addition of olive oil (Figure 1A,C). Olive oil was used to dissolve all the extracts in subsequent experiments. Next, to evaluate time points after administration of the extracts, mice were orally administered the *C. obtusa*, *P. orientalis*, and *J. sabina* extracts for 5 or 10 min followed by a subsequent BaCl_2_ injection. The results revealed no protective effect at 5 min after administration (Figure 1B), while 10 min prior to BaCl_2_ injection was found to have a protective effect (Figure 1D). To investigate the protective effect of amiodarone (clinical medicine as a reference), mice were i.p. injected with 0.6% amiodarone (15 mg/kg Bwt) for 10 min before BaCl_2_ injection, and the ECG signals of the mice appeared normal (Figure 1E). The results further illustrated that BaCl_2_-induced arrhythmia in mice is remediable via clinical drugs. Subsequently, considering the intestinal absorption of the extracts, the conifer plant extract was intragastrically administered for 10 min, followed by an injection of the BaCl_2_ solution. After seven conifer plants were screened, *F. hodginsii*, *J. chinensis* (L.) Ant., *S. chinensis* (L.) Ant. cv. Kaizuca, and *J. chinensis* showed no protective effects against BaCl_2_-induced arrhythmia in mice. Surprisingly, the ECG data revealed protective effects of *C. obtusa*, *P. orientalis*, and *J. sabina* against BaCl_2_-induced arrhythmia in mice (Figure 2, Figure 3 and Figure 4).

### 2.3. Dose-Dependent Protective Effects of C. obtusa, P. orientalis, and J. sabina Extracts on BaCl_2_-Induced Arrhythmia in Mice

To determine the antiarrhythmic effects of *C. obtusa*, *P. orientalis*, and *J. sabina* on BaCl_2_-induced arrhythmia in mice, various doses of *C. obtusa*, *P. orientalis*, *and J. sabina* extracts were tested. The data revealed dose-dependent protective effects of the *C. obtusa*, *P. orientalis*, and *J. sabina* extracts, ranging from 0.075 mL/10 g Bwt to 0.15 mL/10 g Bwt or 0.2 mL/10 g Bwt, while 0.05 mL/10 g Bwt had no significant effect (Figure 2, Figure 3 and Figure 4).

### 2.4. Efficacy of C. obtusa, P. orientalis, and J. sabina Extracts in Protection against BaCl_2_-Induced Arrhythmia in Mice

To investigate the protective potential (duration) of BaCl_2_-induced cardiac arrhythmia, mice were orally administered various doses of *C. obtusa*, *P. orientalis*, and *J. sabina* extracts for 10 min, followed by an injection of the BaCl_2_ solution. The results showed that with increasing doses of *P. orientalis*, and *J. sabina* extracts, the duration of protection increased with increasing dosage. However, due to the toxicity of *C. obtusa*, the duration of protection increased to 0.15 mL/10 g Bwt (Table 1). These results revealed that *C. obtusa*, *P. orientalis*, and *J. sabina* extracts contain antiarrhythmic characters.

### 2.5. Treated Efficacy of C. obtusa, P. orientalis, and J. sabina Extracts in BaCl_2_-Induced Arrhythmia in Rats

**Figure 5:**
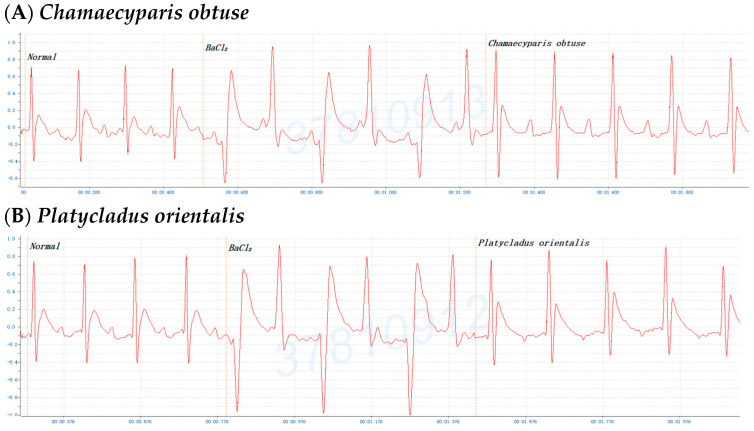

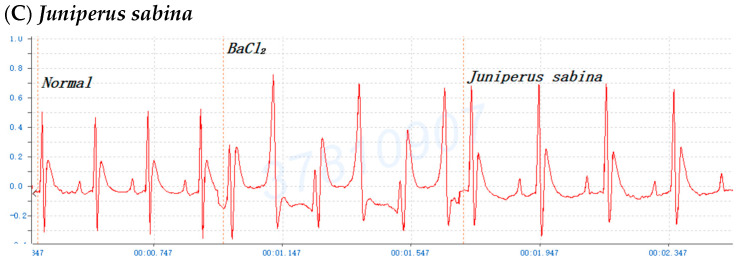
Treatment with 0.4 mL/100 g Bwt of (**A**) *Chamaecyparis obtusa*, (**B**) *Platycladus orientalis*, and (**C**) *Juniperus sabina* in rats after BaCl_2_-induced arrhythmia.

One report indicated that the ethanol extract of *Sophora flavescens* Ait. has been shown to have antiarrhythmic activity via investigations of cardiac arrhythmias induced by aconitine infusion in mice and by coronary artery ligation in rats [38]. To confirm the potential of BaCl_2_-induced cardiac arrhythmia treatment with *C. obtusa*, *P. orientalis*, and *J. sabina* extracts, rats were orally injected with 0.4 mL/100 g Bwt of *C. obtusa*, *P. orientalis*, and *J. sabina* extracts, respectively, of the BaCl_2_ solution. The results revealed the antiarrhythmic effects of *C. obtusa*, *P. orientalis*, and *J. sabina* extracts against BaCl_2_-induced cardiac arrhythmia in rats (Figure 5). These results were consistent with the results obtained in mice, illustrating that *C. obtusa*, *P. orientalis*, and *J. sabina* extracts possessed antiarrhythmic effects.

### 2.6. The Involvement of the M Receptor in the Antiarrhythmic Effects of C. obtusa, P. orientalis, and J. sabina Extracts Compared with That of Amiodarone in Mice

**Figure 6:**
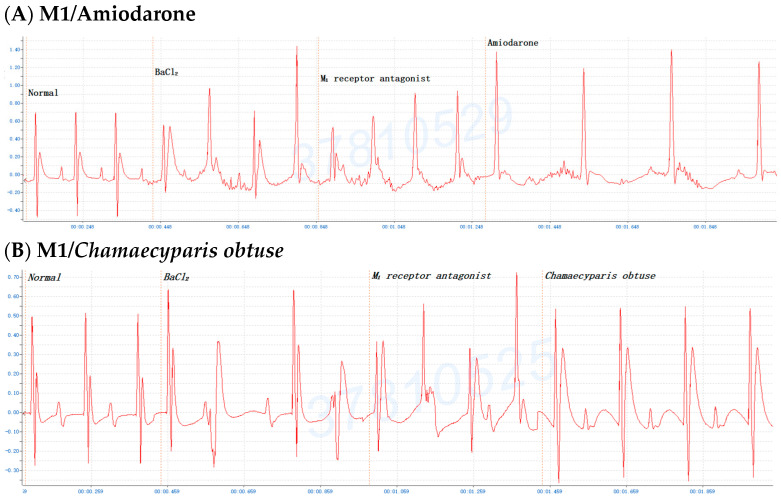

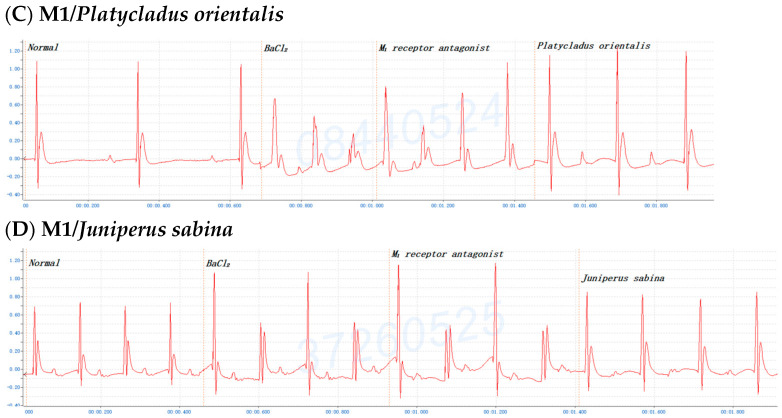
The addition of pirenzepine dihydrochloride (muscarinic receptor M1 antagonist, 0.3 mg/kg) to (**A**) amiodarone or the ethanolic extract (0.1 mL/10 g Bwt) of (**B**) *C. obtusa*, (**C**) *P. orientalis*, and (**D**) *J. sabina* leaves in mice after BaCl_2_-induced arrhythmia.

**Figure 7:**
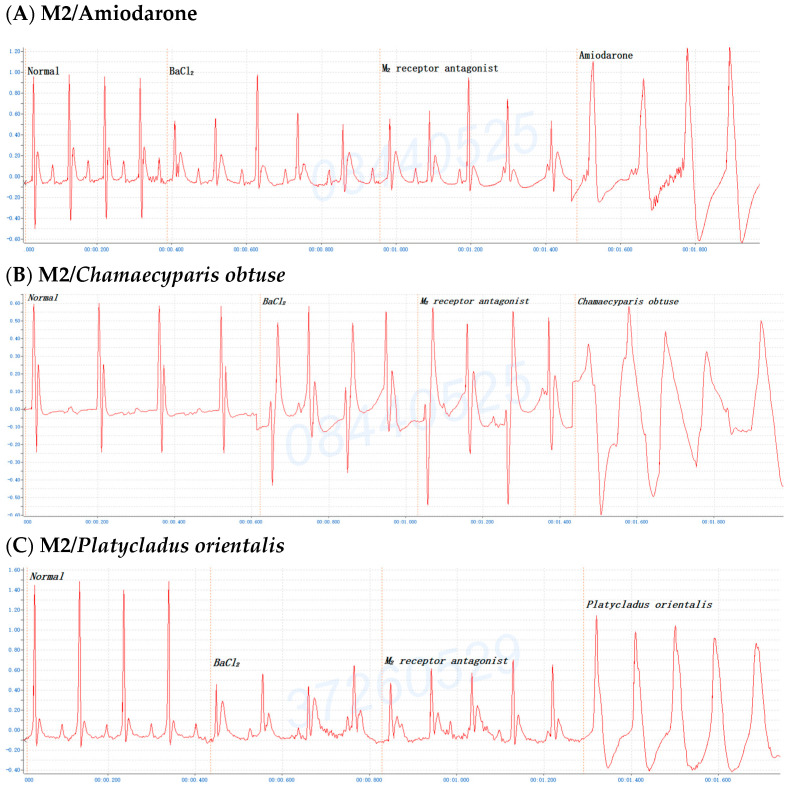

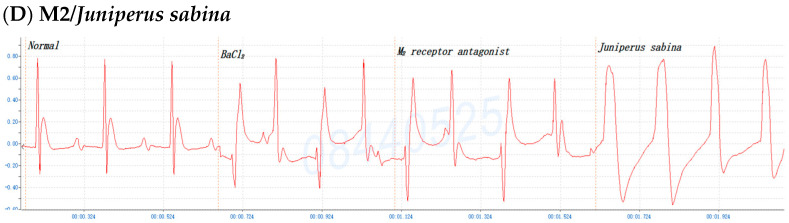
The addition of methoctramine tetrahydrochloride (M2 antagonist, 0.3 mg/kg) with (**A**) amiodarone or ethanolic extract (0.1 mL/10 g Bwt) to (**B**) *C. obtusa*, (**C**) *P. orientalis*, and (**D**) *J. sabina* leaves in mice after BaCl_2_-induced arrhythmia.

**Figure 8:**
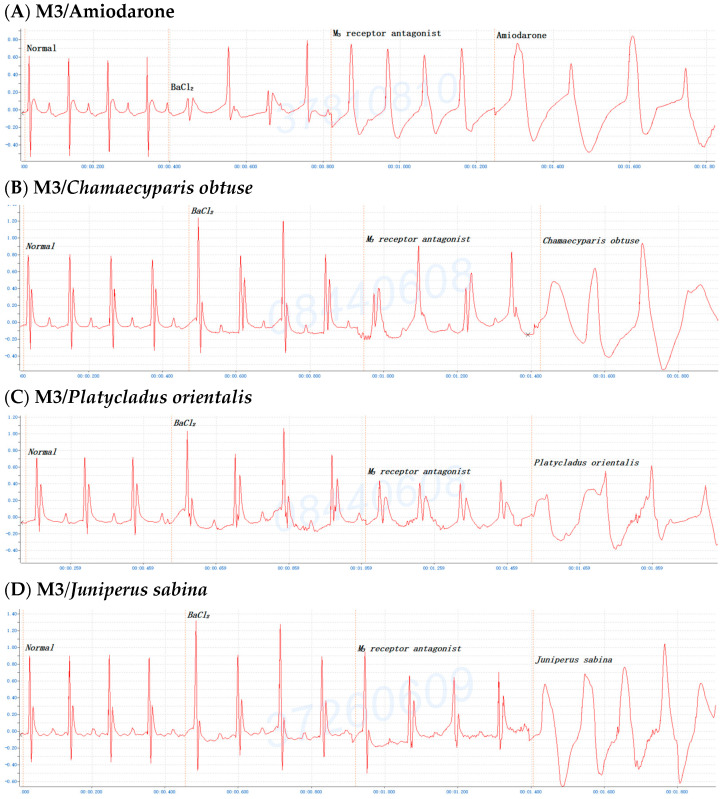
The addition of 4-DAMP (1,1-dimethyl-4-diphenylacetoxypiperidinium iodide, an M3 antagonist, 0.2 mg/kg) to (**A**) amiodarone or the ethanolic extracts (0.1 mL/10 g Bwt) of (**B**) *C. obtusa*, (**C**) *P. orientalis*, and (**D**) *J. sabina* leaves after BaCl_2_-induced arrhythmia in mice.

To further understand which M-receptor type was involved in the antiarrhythmic effects of the *C. obtusa*, *P. orientalis*, or *J. sabina* extracts compared with the reference drug amiodarone, mice were first administered BaCl_2_; once arrhythmia developed, the mice received pirenzepine dihydrochloride (M1 antagonist, 0.3 mg/kg Bwt), methoctramine tetrahydrochloride (M2 antagonist, 0.3 mg/kg Bwt), or 4-DAMP (M3 antagonist, 1 mg/kg Bwt) for 2 min and then amiodarone, *C. obtusa*, *P. orientalis*, or *J. sabina* extracts (0.1 mL/10 g Bwt) to monitor alterations in the ECG. The results demonstrated that the antiarrhythmic effects of the *C. obtusa*, *P. orientalis*, and *J. sabina* extracts could occur through the M2 (Figure 7) and M3 receptors (Figure 8) but not the M1 receptor (Figure 6).

## 3. Discussion

In the present study, we first demonstrated the pharmacological effects of *C. obtusa*, *P. orientalis*, and *J. sabina* extracts in a concentration-dependent manner as the cardioprotective agents against BaCl_2_-induced arrhythmia. As we know, traditional Chinese medicine (TCM) has played a critical role in ameliorating symptoms, thwarting disease recurrence, reducing toxic side effects, and improving the quality of life. The use of Western medicine for the management of AF is limited, and several studies have revealed that traditional herbs comprise a variety of pharmacologically active constituents that have great efficacy and prospective for the hindrance and treatment of cardiac arrhythmia [39,40,41,42] with distinctive advantages, such as few side and adverse effects, low toxicity, a long effect duration, and high compliance. In terms of clinical usage, TCMs with alkaloids, flavonoids, and saponins as the leading effective constituents have a positive effect on the treatment of CVDs such as angina pectoris, arrhythmia, myocardial ischemia, and myocardial infarction (MI) [43]. Another report revealed that Fuzi, *Aconiti lateralis Radix Praeparata*, has been broadly used for 2000 years in TCM for the treatment of acute HF. Notably, the results revealed that the long-term use of Fuzi has a main benefit in averting cardiovascular problems [44]. Calycosin and its derivatives, the major bioactive flavonoids in *Astragalus membranaceus*, have promising potential for the cardiovascular protection of cardiac myocytes and vascular endothelial cells [43]. Wenxin Keli (WXKL), a typical Chinese patent medicine with apparent effectiveness and promising safety, has played a prominent role in the treatment of CVD patients. Accumulating evidence from various cell and animal studies has shown that WXKL plays cardioprotective roles by impeding inflammation, diminishing oxidative stress, mediating vasomotor disorders, decreasing cellular apoptosis, and protecting against endothelial injury, myocardial ischemia, cardiac fibrosis, and cardiac hypertrophy [45]. Furthermore, the action of WXKL may reduce the QT interval and dawdle the heart rate by downregulating sodium channel protein type 5 subunit alpha (SCN5A) and the beta-2 adrenergic receptor (ADRB2) and upregulating muscarinic acetylcholine receptor M2 (CHRM2) during myocardial ischemia. These findings afford novel insight into the molecular mechanisms by which WXKL reduces the prevalence of ventricular arrhythmia [46].

The results of this study disclose that the pharmacological effects of *C. obtusa*, *P. orientalis*, and *J. sabina* extracts exert cardioprotection against BaCl_2_-induced (inhibiting IK1) arrhythmia. Basal and acetylcholine-gated inward-rectifier K^+^-currents (IK1 and IKACh, respectively) play vital roles in cardiac excitability by mediating heart rate variability and susceptibility to atrial arrhythmias and AF. Recent studies have indicated the coexistence of multiple muscarinic acetylcholine receptor (mAChR) subtypes that regulate several distinct K^+^ currents in the heart, namely, the inward rectifier K^+^ current (IKACh) by M2 and two delayed rectifier K^+^ currents, IKM3 and IK4AP, by the M3 and M4 receptors, respectively. Gi-protein-coupled muscarinic receptor M2 is considered the predominant receptor that activates IKACh [47]. Calcium/calmodulin-dependent protein kinase II (CaMKII) is a vital ion channel mediator that participates in excitation–contraction coupling to regulate its electrophysiological function. These effects can be largely abolished by the co-application of the IK1 blocker BaCl_2_. IK1-dependent suppression of CaMKII activity is a crucial cardiac salvage signaling pathway during Ca^2+^ dyshomeostasis and oxidative stress (reactive oxygen species, ROS). IK1 might be a unique target for the pharmacological conditioning of reperfusion arrhythmia, especially during intervention after unpredictable ischemia [48].

Cardiac autonomic nerve dysfunctions, such as the excitement of the Vagal nerve and inhibition of sympathetic nerves, have been exposed by molecular biology studies [49,50]. Accordingly, arrhythmia can be triggered by the abnormal structure and function of ion channels [51]. Accumulating studies have demonstrated a role for α1-adrenolytics in the management of arrhythmias. The stimulation of α1-adrenoceptor facilitates inositol trisphosphate (IP_3_) production and subsequent Ca^2+^ release from the sarcoplasmic reticulum (SR) [52]. Therefore, the blockade of α1-adrenoceptors may result in the stabilization of Ca^2+^ levels, generating antiarrhythmic effects in catecholamine-induced arrhythmias, e.g., catecholaminergic polymorphic ventricular tachycardia. One study has reported that prazosin not only reduced the norepinephrine-induced elongation of AF in mice but also mitigated norepinephrine-induced SR Ca^2+^ leakage and spontaneous SR Ca^2+^ release in cultured atrial cardiomyocytes. These findings confirm that α1-adrenoceptors may have a role in preventing cardiac arrhythmias [53] and have been confirmed in numerous animal studies, thus validating the antiarrhythmic properties of α1-adrenolytics [54,55,56]. In earlier experiments, 2-methoxyphenylpiperazine derivatives were shown to have a high affinity for α1-adrenoceptors [57], and the activities of these compounds were compared with those of carvedilol, which is a β1- and α1-adrenoceptor blocker with antioxidant properties [58]. Nevertheless, the mechanism by which *C. obtusa*, *P. orientalis*, and *J. sabina* extracts protect against BaCl_2_-induced arrhythmia requires verification.

The experiments also depict the involvement of the M receptor in the antiarrhythmic effects of *C. obtusa*, *P. orientalis*, and *J. sabina* extracts compared with that of amiodarone in mice. In primary tissues, at least four pharmacological M receptors (M1, M2, M3, M4) are defined, and five muscarinic receptors have been cloned (m1, m2, m3, m4, m5). There are few selective agonists for M-receptor subtypes, and all classical agonists (acetylcholine, carbachol, etc.) are evidently nonselective. Several selective antagonists for M1 (pirenzepine) and M2 receptors (AF-DX 116) have been critically studied [59]. A comparative study of the ability of selective M-cholinoblockers to prevent arrhythmias induced by phosphacol and potassium cyanide showed that the activity of the M1 antagonist pirenzepine is greater than that of the M2 antagonist AF-DX-116; simultaneously, both drugs revealed the equivalent activity with regard to arrhythmias induced by aconitine, calcium chloride, and carbachol [60]. The present results demonstrated that the antiarrhythmia effects of *C. obtusa*, *P. orientalis*, and *J. sabina* extracts could occur through the M2 (Figure 7) and M3 receptors (Figure 8) but not the M1 receptor (Figure 6). One study has reported that (1) the stimulation of the M1 mAChR subtype on sympathetic postganglionic cells results in catecholamine-mediated cardiac stimulation, (2) M1 mAChR is not expressed in the mouse heart, and (3) the administration of ACh to mice induces arrhythmia [61]. The IKACh plays a vital role in cardiac excitability by mediating heart rate variability and vulnerability to atrial arrhythmias. Both inward rectification mechanisms are extrinsic to the KACh channel; from our understanding, this is the first depiction of an extrinsic inward rectification of ionic current attributable to an intrinsic voltage-sensitive property of the G protein-coupled receptor M2 [62]. Recent studies have indicated the presence of multiple mAChR subtypes that regulate several distinct K+ currents in the heart, namely, the IKACh by M2 and the two delayed rectifier K+ currents IKM3 and IK4AP by the M3 and M4 receptors, respectively [63]. This is the first report to demonstrate the downregulation of three types of mAChR-coupled K^+^ currents (IKM2, IKM3, and IKM4) and of M2, M3, and M4 mAChR subtype proteins in a canine model of atrial tachycardia (AT)-induced remodeling [64]. Both D,L-sotalol and MS-551 interact with cardiac M2 and peripheral M3 receptors, and they exert anticholinergic activity in cardiac and peripheral tissues at high concentrations [65]. Activation of M3 has been previously shown to promote short-term cardioprotection against ischemic injury with the M3 agonist choline, the antagonist 4-DAMP, or the M2-mAChR antagonist methoctramine followed by the administration of choline. M3-mAChRs denote a promising target for interpreting cardiomyocytes tolerant to ischemic injury [66]. The prevention of ischemia-induced changes in Gi-mediated signal transduction and/or (with certain limitations discussed below) the selective activation of cardiac muscarinic M2 receptors could hence be an alternative pharmacological treatment for acute myocardial ischemia [67]. Atrial G protein-gated inwardly rectifying K+ (GIRK) channels are critical mediators of parasympathetic effects on cardiac physiology. The mouse ventricular GIRK channel is a GIRK channel subunit (GIRK1, GIRK4), a GIRK1/GIRK4 heteromer, and although it contributes to the cholinergic suppression of ventricular myocyte excitability, this impact does not substantively influence cardiac physiology or ventricular arrhythmogenesis in mice [68]. Accumulating evidence indicates the presence of functional M3-mAChRs, in addition to the well-recognized M2-mAChRs, in the hearts of various species comprising humans. Choline improves cardiac function and moderates ischemic myocardial injury by stimulating cardiac M3-mAChRs, which in turn results in changes in the multiple signaling pathways, leading to cytoprotection. These findings suggest that M3-mAChR is a new target for improving cardiac function and preventing cardiac injury during ischemia/reperfusion [69].

Remarkably, bioactive compounds such as phenolics, flavonoids, terpenoids, glycosidic derivatives, alkaloids, iridoids, and saponins from various parts of plants including *Terminalia arjuna* [70] and *Emblica officinalis* fruit [71] have gained important applications in exerting significantly cardioprotective effects. Furthermore, many lines of medicinal values have been discovered from *Cupressus sempervirens* L. (Cupressaceae) including headache, toothaches [72], sneeze bronchitis, obesity, and laryngitis along with biological properties such as anti-inflammatory, anti-microbial, and insecticidal actions [73]. In addition, nineteen various polyphenolic molecules comprising gallic acid, chlorogenic acid, catechin, methyl gallate, coffeic acid, syringic acid, pyrocatechol, rutin, ellagic acid, coumaric acid, vanillin, ferulic acid, naringenin, rosmarinic acid, daidzein, querectin, cinnamic acid, kaempferol, and hesperetin upon extraction of *C. sempervirens* using supercritical fluid extraction have recently been identified and further been investigated for their antibacterial and anti-biofilm activities [74,75]. To the best of our knowledge, this study is the first investigation to explore the potential benefit of Cupressaceae in the treatment of arrhythmia, making our findings particularly valuable. In the future, we will be treating large animal arrhythmia with the extracts to confirm the efficacy of ethanolic extracts. And the extracts are isolated and purified to obtain the active components via HPLC and NMR. The experiments will further be conducted to investigate the purified components in in vivo and in vitro assays in the treatment of animal arrhythmia to validate the efficacy, acting mechanisms, and toxicity for the completion of preclinical tests.

## 4. Materials and Methods

### 4.1. Plant Source and Reagents

Conifer plants (*F. hodginsii* and *C. obtusa*) were collected from Xiamen (Fujian, China), and others (*J. chinensis* (L.) Ant., *S. chinensis* (L.) Ant. cv. Kaizuca, *P. orientalis* (L.) Franco, *J. sabina* L., and *J. chinensis* ‘Pyramidalis’) were obtained from Jiangsu (China). The experimental plant samples, including the collected plant material, complied with relevant institutional, national, and international guidelines. Leaves of cypress varieties were cleaned thoroughly with water and rinsed with distilled water. The plant materials were dried under shade at room temperature (RT). All chemicals were purchased from Sigma–Aldrich (St. Louis, MO, USA). Pirenzepine dihydrochloride (muscarinic receptor M1 antagonist), methoctramine tetrahydrochloride (M2 antagonist), and 4-DAMP (1,1-dimethyl-4-diphenylacetoxypiperidinium iodide, M3 antagonist) were obtained from MedChemExpress (Shanghai, China).

### 4.2. Extraction of Cypress Leaves

First, all the dried cypress leaves were cut into small pieces. Second, all the cut leaves were soaked in 75% ethanol (1:10, *w*/*v*) in an ultrasonic bath (KQ-5200 type DE, Kun Shan Ultrasonic Instruments Co., Ltd., Kunshan, Jiangsu, China) to obtain the extraction solutions. Finally, the extraction solutions were dried in an evaporator (DHG-9070A, Shanghai Heheng Instruments and Equipment Co., Ltd., Shanghai, China) at 42 °C to obtain the products as dark green solids. The extracts were then dissolved in olive oil for subsequent experiments. The yield of crude extract was 8.6 ± 1.2%. The experimental concentration was 40 mg/mL.

### 4.3. Animal Care

All experimental procedures were executed according to the guidelines outlined in the “Guide for the Care and Use of Laboratory Animals, 8th edition” published by the National Institutes of Health, and they fulfilled the ARRIVE guidelines. The animal experiments were approved by the Animal Ethics Committee of Medical College according to the “Guide for the Care and Use of Laboratory Animals” of Xiamen Medical College (approved protocol ID SYXK 2018-0010). Evaluations of experimental animal care were periodically performed in accordance with the Laboratory Animal Guidelines for Ethical Review of Animal Welfare (GB/T 35892-2018, China).

Eighty male ICR mice (6 weeks old, 22 ± 3 g Bwt) and male Sprague Dawley rats (150 ± 30 g Bwt) were obtained from Hangzhou Medical College (Zhejiang, China) and kept at RT (22 ± 2 °C) and a specific humidity (50 ± 10%). A 12/12 h light/dark (6 a.m.–6 p.m.) cycle was maintained throughout the entire study. The mice had free access to a standard laboratory diet (Rodent Feed 1022, Beijing HFK Bioscience Co., Ltd., Beijing, China) and tap water ad libitum. Sprague Dawley rats and ICR mice were anesthetized with 5% isoflurane gas in an inhalation chamber with a vaporizer (R583S rodent gas anesthesia machine, RWD Life Science Co., Ltd., Shenzhen, China), and 2% isoflurane was administered during the entire experimental procedure. The mice were anesthetized according to the protocol supplied in the McGill Standard Operating Procedure (SOP) (#110 for mice and #111 for rats), which describes methods used for mouse and rat anesthesia.

### 4.4. Antiarrhythmic Activity of the Extract in a Mouse Model of BaCl_2_-Induced Arrhythmia Prior to (Protection) BaCl_2_ Induction

Mice were randomly divided into 9 groups: an NS group, a positive control group (amiodarone), and 7 test groups of Cupressaceae leaves. First, the mice were generally anesthetized with 5% inhaled isoflurane in a rodent gas anesthesia machine (RWD Life Science Co., Ltd. Shenzhen, China) and fixed on a plank. Second, acupuncture needles were subcutaneously inserted into the limbs of the mice to monitor and record normal II lead electrocardiograms (ECGs) via the BL-420I biological function experiment system (Techman Inc., Chengdu, Sichuan, China) under 2% isoflurane anesthesia. Then, the anesthetized mice were orally administered different treatments at the test dose. Ten minutes later, a 0.8% barium chloride solution was i.p. injected into each mouse (0.1 mL/10 g Bwt) to induce arrhythmia. ECG signals were individually monitored and recorded during the experiments. The number of mice that maintained a normal rhythm and the duration (5, 10, and 30 min) of normal rhythm mice were recorded to calculate the efficacy of the tested samples in each group. At the end of the experiments, all rats were sacrificed by CO_2_ euthanasia. Additional experiments were performed to explore the effect of the muscarinic acetylcholine receptor type on the antiarrhythmic effects of the *C. obtusa*, *P. orientalis*, and *J. sabina* extracts (5 mg/mL of olive oil). Mice were treated with pirenzepine dihydrochloride (an M1 antagonist, 0.3 mg/kg Bwt), methoctramine tetrahydrochloride (an M2 antagonist, 0.3 mg/kg Bwt), or 4-DAMP (an M3 antagonist, 1 mg/kg Bwt) for 2 min and administered amiodarone, *C. obtusa*, *P. orientalis*, or *J. sabina* extracts (0.1 mL/10 g Bwt) to detect alterations in the ECG profile.

### 4.5. Statistical Analysis

The in vivo data are expressed as means ± SEMs. The results were carried out by using a one-way analysis of variance (ANOVA) for statistical comparisons among treatments. The means within each column followed by different letters are significantly different at *p* < 0.05 according to the post hoc Tukey’s test.

## 5. Conclusions

CVDs are the leading cause of global mortality and impose a considerable economic burden on both governments and individuals. Ethanolic extracts of seven species of Cupressaceae, namely, *Chamaecyparis obtusa*, *Juniperus chinensis* (L.) Ant., *Sabina chinensis* (L.) Ant. cv. Kaizuca, *Platycladus orientalis* (L.) Franco, *Juniperus sabina* L., *Fokienia hodginsii*, and *Juniperus chinensis* ‘Pyramidalis’ were screened for their cardioprotective effects against BaCl_2_-induced arrhythmia in a mouse model, and the results of the ECG profiles revealed that pretreatment with *C. obtusa*, *P. orientalis*, and *J. sabina* extracts exerted dose-dependent cardioprotective effects. The cardioprotection of the *C. obtusa*, *P. orientalis*, and *J. sabina* extracts was exerted through the M2- and M3-mAChRs. These treatment effects were also confirmed in a rat model. These first-line key findings reveal that certain Cupressaceae species possess active antiarrhythmic components, suggesting that these innovative phytocompounds might have promising potential for preventing the occurrence of cardiac arrhythmia and reducing SCD. The identification of active phytochemicals and their antiarrhythmic implications in the clinic requires further study.

## Figures and Tables

**Table 1:** The protection duration (min) of barium chloride-induced cardiac arrhythmia pretreated with the ethanol extracts of *Chamaecyparis obtusa*, *Platycladus orientalis*, and *Juniperus sabina* leaves.

Species	0.05 mL/10 g Bwt	0.075 mL/10 g Bwt	0.10 mL/10 g Bwt	0.15/0.20 mL/10 g Bwt
***Chamaecyparis obtusa***	0.5 ± 0.2 min	4.0 ± 1.1 min **	5.0 ± 1.1 min **	15.0 ± 2.9 min *** (0.15 mL/10 g)
***Platycladus orientalis***	1.0 ± 0.4 min	5.0 ± 1.4 min **	5.0 ± 1.7 min **	9.0 ± 2.1 min **
***Juniperus sabina***	0.5 ± 0.3 min	4.5 ± 1.9 min **	5.0 ± 1.5 min **	10.0 ± 2.3 min ***

Mice was orally pretreated with various concentrations of ethanol extracts of *C. obtusa*, *P. orientalis*, or *J. sabina* leaves (each dose *n* = 6) for 10 min followed by a 0.8% barium chloride (0.1 mL/10 g Bwt) injection (i.p.). ** *p* < 0.01 and *** *p* < 0.005 compared with 0.05 mL/10 g Bwt.

## Data Availability

Data are contained within the article and Supplementary Materials.

## Supplementary Materials

The following supporting information can be downloaded at: https://www.mdpi.com/article/10.3390/ph17081003/s1, Table S1: The chemo-information and biological functions of Cupressaceae species.

## Abbreviations

AADs (antiarrhythmic drugs); ADRB2 (beta-2 adrenergic receptor); AT (atrial tachycardia); BaCl_2_ (barium chloride); CaMKII (calcium/calmodulin-dependent protein kinase II); CHRM2 (muscarinic acetylcholine receptor M2); *C. obtuse (Chamaecyparis obtusa)*; CVDs (cardiovascular diseases); DAMP (1,1-dimethyl-4-diphenylacetoxypiperidinium iodide); ECG (electrocardiogram); GIRK (Atrial G protein-gated inwardly rectifying K^+^); HF (heart failure); IKACh (acetylcholine (ACh)-gated inwardly rectifying K+ current); IP_3_ (inositol trisphosphate); *J. sabina* (*Juniperus sabina*); mAChRs (muscarinic acetylcholine receptor subtypes); MI (myocardial infarction); *P. orientalis (Platycladus orientalis*); ROS (reactive oxygen species); SCD (sudden cardiac death); SCN5A (5 subunit alpha); SOP (standard operating procedure); SR (sarcoplasmic reticulum); TCM (traditional Chinese medicine); WXKL (Wenxin Keli).

## References

[1] Krittayaphong R., Rangsin R., Thinkhamrop B., Hurst C., Rattanamongkolgul S., Sripaiboonkij N., Yindeengam A. (2016). Prevalence and associating factors of atrial fibrillation in patients with hypertension: A nation-wide study. BMC Cardiovasc. Disord..

[2] Bei Y., Shi C., Zhang Z., Xiao J. (2019). Advance for Cardiovascular Health in China. J. Cardiovasc. Transl. Res..

[3] Kusumoto F.M., Bailey K.R., Chaouki A.S., Deshmukh A.J., Gautam S., Kim R.J., Kramer D.B., Lambrakos L.K., Nasser N.H., Sorajja D. (2018). Systematic review for the 2017 AHA/ACC/HRS guideline for management of patients with ventricular arrhythmias and the prevention of sudden cardiac death: A Report of the American College of Cardiology/American Heart Association Task Force on Clinical Practice Guidelines and the Heart Rhythm Society. Heart Rhythm..

[4] Wang W., Cadrin-Tourigny J., Bhonsale A., Tichnell C., Murray B., Monfredi O., Chrispin J., Crosson J., Tandri H., James C.A. (2018). Arrhythmic outcome of arrhythmogenic right ventricular cardiomyopathy patients without implantable defibrillators. J. Cardiovasc. Electrophysiol..

[5] Habibi M., Berger R.D., Calkins H. (2021). Radiofrequency ablation: Technological trends, challenges, and opportunities. Europace.

[6] Sandhu A., Levy A., Varosy P.D., Matlock D. (2019). Implantable cardioverter defibrillators and cardiac resynchronization therapy in older adults with heart failure. J. Am. Geriatr. Soc..

[7] Al-Khatib S.M., Kusumoto F.M. (2019). Implantable cardioverter-defibrillators have stood the test of time!. Circulation.

[8] Honarbakhsh S., Hunter L., Chow A., Hunter R.J. (2018). Bradyarrhythmias and pacemakers. BMJ.

[9] Fuchs M., Schibilsky D., Zeh W., Berchtold-Herz M., Beyersdorf F., Siepe M. (2019). Does the heart transplant have a future?. Eur. J. Cardio-Thorac. Surg..

[10] Sutanto H., Laudy L., Clerx M., Dobrev D., Crijns H.J.G.M., Heijman J. (2019). Maastricht antiarrhythmic drug evaluator (MANTA): A computational tool for better understanding of antiarrhythmic drugs. Pharmacol. Res..

[11] Lu J., Jones A.D., Harkema J.R., Roth R.A., Ganey P.E. (2012). Amiodarone exposure during modest inflammation induces idiosyncrasy-like liver injury in rats: Role of tumor necrosis factor-alpha. Toxicol. Sci..

[12] Mahavadi P., Henneke I., Ruppert C., Knudsen L., Venkatesan S., Liebisch G., Chambers R.C., Ochs M., Schmitz G., Vancheri C. (2014). Altered surfactant homeostasis and alveolar epithelial cell stress in amiodarone-induced lung fibrosis. Toxicol. Sci..

[13] Brugada J., Katritsis D.G., Arbelo E., Arribas F., Bax J.J., Blomström-Lundqvist C., Calkins H., Corrado D., Deftereos S.G., Diller G.P. (2020). 2019 ESC Guidelines for the management of patients with supraventricular tachycardia. The Task Force for the management of patients with supraventricular tachycardia of the European Society of Cardiology (ESC). Eur. Heart J..

[14] Yang N.J., Liu Y.R., Tang Z.S., Duan J.A., Yan Y.F., Song Z.X., Wang M.G., Zhang Y.R., Chang B.J., Zhao M.L. (2021). Poria cum Radix Pini Rescues Barium Chloride-Induced Arrhythmia by Regulating the cGMP-PKG Signalling Pathway Involving ADORA1 in Zebrafish. Front. Pharmacol..

[15] Wink M. (2015). Modes of Action of Herbal Medicines and Plant Secondary Metabolites. Medicines.

[16] Huyan T., Li Q., Wang Y.L., Li J., Zhang J.Y., Liu Y.X., Shahid M.R., Yang H., Li H.Q. (2016). Anti-tumor effect of hot aqueous extracts from *Sonchus oleraceus* (L.) L. and *Juniperus sabina L*—Two traditional medicinal plants in China. J. Ethnopharmacol..

[17] Abdel-Kader M.S., Soliman G.A., Alqarni M.H., Hamad A.M., Foudah A.I., Alqasoumi S.I. (2019). Chemical composition and protective effect of *Juniperus sabina,* L. essential oil against CCl4 induced hepatotoxicity. Saudi Pharm. J..

[18] Xu S., Li X., Liu S., Tian P., Li D. (2022). *Juniperus sabina,* L. as a Source of Podophyllotoxins: Extraction Optimization and Anticholinesterase Activities. Int. J. Mol. Sci..

[19] Orhan N., Deliorman Orhan D., Gökbulut A., Aslan M., Ergun F. (2017). Comparative Analysis of Chemical Profile, Antioxidant, In-vitro and In-vivo Antidiabetic Activities of *Juniperus foetidissima* Willd. and *Juniperus sabina* L.. Iran. J. Pharm. Res..

[20] Shi R., Li J. (2022). Study on the mechanism of *Sabina przewalskii* on chronic obstructive pulmonary disease based on network pharmacology. J. Qinghai Norm. Univ..

[21] Park S.A., Jegal J., Chung K.W., Jung H.J., Noh S.G., Chung H.Y., Ahn J., Kim J., Yang M.H. (2018). Isolation of tyrosinase and melanogenesis inhibitory flavonoids from *Juniperus chinensis* fruits. Biosci. Biotechnol. Biochem..

[22] Cui Y., Nan P., Lin M. (2006). Main Volatile Components in the Leaves of *Sabina chinensis* L. Ant. and *Sabina chinensis* L. Ant. Cv. Kaizuca and Their Effects on Bacteria. J. Environ. Health.

[23] Andersen F.A. (2001). Final report on the safety assessment of *Juniperus communis* Extract, *Juniperus oxycedrus* Extract, *Juniperus oxycedrus* Tar, *Juniperus phoenicea* Extract, and *Juniperus virginiana* Extract. Int. J. Toxicol..

[24] Li R.L., Wu P.L., Li C.R. (2019). Study on the chemical constituents of the branches and leaves of *Juniperus formosana*. West China J. Pharm..

[25] Wu S., Lin Y. (2014). Advances in Chinese Juniper leaves. Chin. J. Ethn. Med..

[26] Jiang J.H., Li X.C., Gao T.H., He D.N., Chen F.M., Zhang Y.P., Huang L.B. (2006). Volatile Constituents from the Cupressaceae Plants and Their Antitumor Activities. J. Fujian For. Sci. Technol..

[27] Wu X.D., He J., Li X.Y., Dong L.B., Gong X., Song L.D., Li Y., Peng L.Y., Zhao Q.S. (2013). Diterpenoids from the twigs and leaves of *Fokienia hodginsii*. J. Nat. Prod..

[28] Wu X.D., Zhong W.W., Ding L.F., Tu W.C., Yang H., Gong X., Peng L.Y., Li Y., Xu Z.Z., Zhao Q.S. (2017). Sesquiterpenoids from the twigs and leaves of *Fokienia hodginsii*. J. Asian Nat. Prod. Res..

[29] Hau D.V., Sa N.H., Tam N.T., Diep N.T., Hoang Anh N.T., Thuy Linh N.T., Ngoc Ni H.T., Adorisio S., Delfino D.V., Thuy T.T. (2021). Pro-apoptoticeffect of diterpenoids from *Fokienia hodginsii* on acute myeloid leukemia cells. Nat. Prod. Res..

[30] Zhang Y., Yang S., Cao Q., Zhang W., Chen F. (2008). Study on the Chemical Constituents and Biological Activities of Volatile Oil from *Fokienia hodgisii*. Anhui Agric. Sci..

[31] Manimaran S., Kumar B.S., Khan S., Patel D., Suresh B. (2005). Anti inflammatory activity of cone vola tile oil of *Cupressus funebris* endl. Anc. Sci. Life..

[32] Liu Y.X. (2013). Analysis of Chemical Constituents of Essential Oil Extracted from Leaves and Seeds of *Sabina chinensis* L. J. Hubei Univ. Natl..

[33] Gu D., Fang C., Yang J., Li M., Liu H., Yang Y. (2018). Chemical composition and α-amylase inhibitory activity of the essential oil from *Sabina chinensis* cv. Kaizuca leaves. Nat. Prod. Res..

[34] Zhang J., Zhao Z., Liang W., Bi J., Zheng Y., Gu X., Fang H. (2022). Essential oil from *Sabina chinensis* leaves: A promising green control agent against Fusarium sp.. Front. Plant Sci..

[35] Ibrahim N.A., El-Seedi H.R., Mohammed M.M. (2007). Phytochemical investigation and hepatoprotective activity of *Cupressus sempervirens,* L. leaves growing in Egypt. Nat. Prod. Res..

[36] Leigh-de Rapper S., Viljoen A., van Vuuren S. (2021). Essential Oil Blends: The Potential of Combined Use for Respiratory Tract Infections. Antibiotics.

[37] Hao D., Zhang Y., Dai H., Wang Y. (2006). Analysis of volatile constituents in leaves of three cypress species by gas chromatography/mass spectrometry. Se Pu = Chin. J. Chromatogr..

[38] Dai S., Chan M.Y., Lee S.S., Ogle C.W. (1986). The antiarrhythmic effects of *Sophora flavescens* Ait. in rats and mice. Am. J. Chin. Med..

[39] He H., Han G., Li X., Lan H., Li Y., Dou X., Guo Y., Zhang M., Liu H. (2021). Efficacy and Safety of Chinese Medicine in Treating Arrhythmia: Meta-Analysis of Randomized Controlled Trials. Evid. Based Complement. Alternat Med..

[40] Yang Y., Ge F.L., Huang Q., Zeng R., Zhang X.Y., Liu P., Luo G., Yang S.J., Sun Q. (2022). Randomized Controlled Trials of Zhigancao Decoction Combined with Metoprolol in the Treatment of Arrhythmia: A Systematic Review and Meta-Analysis. Front. Cardiovasc. Med..

[41] Yuan H., Ma Q., Ye L., Piao G. (2016). The Traditional Medicine and Modern Medicine from Natural Products. Molecules.

[42] Bryzgalov A.O., Tolstikova T.G., Shults E.E., Petrova K.O. (2018). Natural Products as a Source of Antiarrhythmic Drugs. Mini Rev. Med. Chem..

[43] Pan L., Zhang X.F., Wei W.S., Zhang J., Li Z.Z. (2020). The cardiovascular protective effect and mechanism of calycosin and its derivatives. Chin. J. Nat. Med..

[44] Tai C.J., El-Shazly M., Yang Y.H., Tsai Y.H., Csupor D., Hohmann J., Wu Y.C., Tseng T.G., Chang F.R., Wang H.C. (2022). The effectiveness of Fuzi in combination with routine heart failure treatment on chronic heart failure patients. J. Ethnopharmacol..

[45] Tian G., Sun Y., Liu S., Li C., Chen S., Qiu R., Zhang X., Li Y., Li M., Shang H. (2018). Therapeutic Effects of Wenxin Keli in Cardiovascular Diseases: An Experimental and Mechanism Overview. Front. Pharmacol..

[46] Yao Y., Liu Y., Zeng Z., Zhao Y., Li T., Chen R., Zhang R. (2020). Identification of Target Genes of Antiarrhythmic Traditional Chinese Medicine Wenxin Keli. Cardiovasc. Ther..

[47] Heijman J., Kirchner D., Kunze F., Chrétien E.M., Michel-Reher M.B., Voigt N., Knaut M., Michel M.C., Ravens U., Dobrev D. (2018). Muscarinic type-1 receptors contribute to IKACh in human atrial cardiomyocytes and are upregulated in patients with chronic atrial fibrillation. Int. J. Cardiol..

[48] Liu Q., Sun J., Zhang L., Xu Y., Wu B., Cao J. (2021). The Agonist of Inward Rectifier Potassium Channel (IK1) Attenuates Rat Reperfusion Arrhythmias Linked to CaMKII Signaling. Int. Heart J..

[49] de Araújo R.B., Azevedo B.M.S., Andrade T.S., Abalem M.F., Monteiro M.L.R., Carricondo P.C. (2018). Subconjunctival 0.1% epinephrine versus placebo in maintenance of mydriasis during vitrectomy: A randomized controlled trial. Int. J. Retin. Vitr..

[50] Janssens U., Michels G. (2019). Adrenalin bei Patienten mit prähospitalem Herz-Kreislauf-Stillstand: PARAMEDIC2-Studie Adrenaline in patients with out-of-hospital cardiac arrest: PARAMEDIC2 trial. Med. Klin. Intensivmed. Notfmed..

[51] Torrente A.G., Mesirca P., Bidaud I., Mangoni M.E. (2020). Channelopathies of voltage-gated L-type Cav1.3/α1D and T-type Cav3.1/α1G Ca2+ channels in dysfunction of heart automaticity. Pflugers Arch..

[52] Escobar G.J., LaGuardia J.C., Turk B.J., Ragins A., Kipnis P., Draper D. (2012). Early detection of impending physiologic deterioration among patients who are not in intensive care: Development of predictive models using data from an automated electronic medical record. J. Hosp. Med..

[53] Suita K., Fujita T., Hasegawa N., Cai W., Jin H., Hidaka Y., Prajapati R., Umemura M., Yokoyama U., Sato M. (2015). Norepinephrine-Induced Adrenergic Activation Strikingly Increased the Atrial Fibrillation Duration through β1- and α1-Adrenergic Receptor-Mediated Signaling in Mice. PLoS ONE..

[54] Sapa J., Kubacka M. (2011). The possible mechanism of hypotensive activity of some pyrrolidin-2-one derivatives with antagonist properties at alpha1-adrenoceptors. Eur. J. Pharmacol..

[55] Kubacka M., Mogilski S., Filipek B., Marona H. (2013). The hypotensive activity and alpha1-adrenoceptor antagonistic properties of some aroxyalkyl derivatives of 2-methoxyphenylpiperazine. Eur. J. Pharmacol..

[56] Rapacz A., Sapa J., Nowiński L., Mogilski S., Pytka K., Filipek B., Siwek A., Szkaradek N., Marona H. (2015). Biofunctional studies of new 2-methoxyphenylpiperazine xanthone derivatives with α₁-adrenolytic properties. Pharmacol. Rep..

[57] Pytka K., Rapacz A., Zygmunt M., Olczyk A., Waszkielewicz A., Sapa J., Filipek B. (2015). Antidepressant-like activity of a new piperazine derivative of xanthone in the forced swim test in mice: The involvement of serotonergic system. Pharmacol. Rep..

[58] Pytka K., Lustyk K., Żmudzka E., Kotańska M., Siwek A., Zygmunt M., Dziedziczak A., Śniecikowska J., Olczyk A., Gałuszka A. (2016). Chemically Homogenous Compounds with Antagonistic Properties at All α1-Adrenoceptor Subtypes but not β1-Adrenoceptor Attenuate Adrenaline-Induced Arrhythmia in Rats. Front. Pharmacol..

[59] van Zwieten P.A., Doods H.N. (1995). Muscarinic receptors and drugs in cardiovascular medicine. Cardiovasc. Drugs Ther..

[60] Kosmachev A.B., Fil’ko O.A., Petrov V.V. (2002). Izuchenie roli otdel’nykh podtipov M-kholinoretseptorov v narushenii serdechnogo ritma razlichnoĭ étiologii the role of M-cholinoreceptor subtypes in heart rhythm disturbances of various etiology. Eksp. Klin. Farmakol..

[61] Hardouin S.N., Richmond K.N., Zimmerman A., Hamilton S.E., Feigl E.O., Nathanson N.M. (2002). Altered cardiovascular responses in mice lacking the M(1) muscarinic acetylcholine receptor. J. Pharmacol. Exp. Ther..

[62] Salazar-Fajardo P.D., Aréchiga-Figueroa I.A., López-Serrano A.L., Rodriguez-Elias J.C., Alamilla J., Sánchez-Chapula J.A., Tristani-Firouzi M., Navarro-Polanco R.A., Moreno-Galindo E.G. (2018). The voltage-sensitive cardiac M2 muscarinic receptor modulates the inward rectification of the G protein-coupled, ACh-gated K+ current. Pflugers Arch..

[63] Shi H., Wang H., Li D., Nattel S., Wang Z. (2004). Differential alterations of receptor densities of three muscarinic acetylcholine receptor subtypes and current densities of the corresponding K+ channels in canine atria with atrial fibrillation induced by experimental congestive heart failure. Cell Physiol. Biochem..

[64] James A.F., Hancox J.C. (2007). More types than one: Multiple muscarinic receptor coupled K+ currents undergo remodelling in an experimental model of atrial fibrillation. Br. J. Pharmacol..

[65] Uemura H., Hara Y., Endou M., Mori K., Nakaya H. (1995). Interaction of class III antiarrhythmic drugs with muscarinic M2 and M3 receptors: Radioligand binding and functional studies. Naunyn Schmiedebergs Arch. Pharmacol..

[66] Zhao J., Su Y., Zhang Y., Pan Z., Yang L., Chen X., Liu Y., Lu Y., Du Z., Yang B. (2010). Activation of cardiac muscarinic M3 receptors induces delayed cardioprotection by preserving phosphorylated connexin43 and up-regulating cyclooxygenase-2 expression. Br. J. Pharmacol..

[67] Rauch B., Niroomand F. (1991). Specific M2-receptor activation: An alternative to treatment with beta-receptor blockers?. Eur. Heart J..

[68] Anderson A., Kulkarni K., Marron Fernandez de Velasco E., Carlblom N., Xia Z., Nakano A., Martemyanov K.A., Tolkacheva E.G., Wickman K. (2018). Expression and relevance of the G protein-gated K+ channel in the mouse ventricle. Sci. Rep..

[69] Yang B., Lin H., Xu C., Liu Y., Wang H., Han H., Wang Z. (2005). Choline produces cytoprotective effects against ischemic myocardial injuries: Evidence for the role of cardiac m3 subtype muscarinic acetylcholine receptors. Cell Physiol. Biochem..

[70] Ramesh P., Palaniappan A. (2023). Terminalia arjuna, a Cardioprotective Herbal Medicine–Relevancy in the Modern Era of Pharmaceuticals and Green Nanomedicine—A Review. Pharmaceuticals.

[71] Hashem-Dabaghian F., Ziaee M., Ghaffari S., Nabati F., Kianbakht S. (2018). A systematic review on the cardiovascular pharmacology of *Emblica officinalis* Gaertn. J. Cardiovasc. Thorac. Res..

[72] Nehdi I.A. (2013). Cupressus sempervirens var. horizentalis seed oil: Chemical composition, physicochemical characteristics, and utilizations. Ind. Crops Prod..

[73] Hasaballah A., Shehata A., Fouda M., Hassan M., Gad M. (2018). The biological activity of *Cupressus sempervirens* extracts against *Musca domestica*. Asian J. Biol..

[74] Mohamed A. (2024). Biological applications of *Cupressus sempervirens* biomass extracted at various levels of pressure using different critical fluid extraction protocol. Biomass Conv. Bioref..

[75] Zhao J., Gu Z., Liu T., Xu F., You S., Li C. (2016). Anti-arthritic effects of total flavonoids from Juniperus sabina on complete freund’s adjuvant induced arthritis in rats. Pharmacogn. Mag..

