# Supplementary material for: Ethanolic Extracts of Cupressaceae Species Conifers Provide Rapid Protection against Barium Chloride-Induced Cardiac Arrhythmia
Source: Pharmaceuticals (Basel). 2024 Jul 29;17(8):1003. doi: 10.3390/ph17081003 (PMC11356987; doi:10.3390/ph17081003)
Supplement: Supplementary file 1 [file pharmaceuticals-17-01003-s001.zip › pharmaceuticals-3074817-supplementary.pptx]

## Slide 1
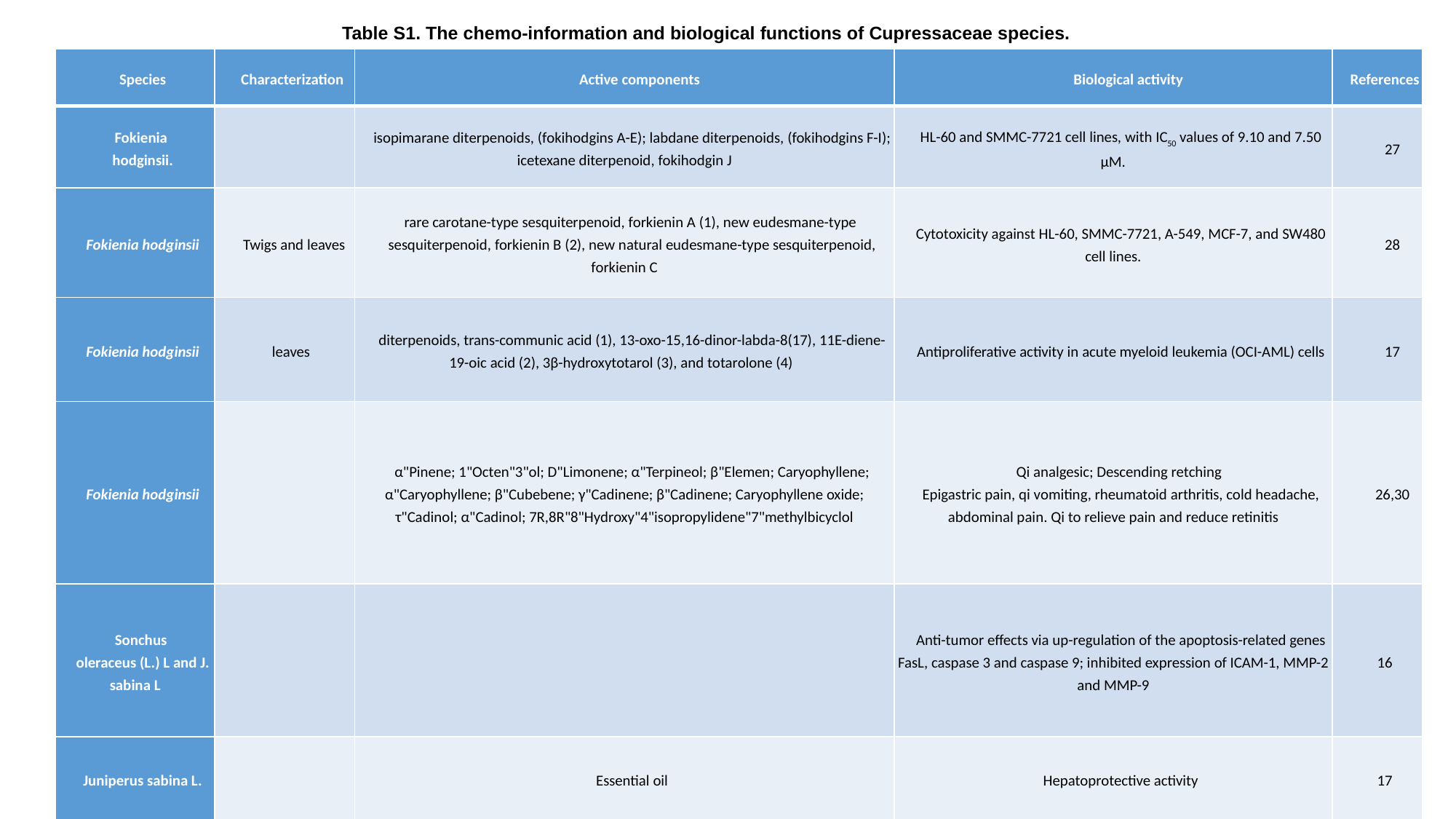

Table S1. The chemo-information and biological functions of Cupressaceae species.
| Species | Characterization | Active components | Biological activity | References |
| --- | --- | --- | --- | --- |
| Fokienia hodginsii. | | isopimarane diterpenoids, (fokihodgins A-E); labdane diterpenoids, (fokihodgins F-I); icetexane diterpenoid, fokihodgin J | HL-60 and SMMC-7721 cell lines, with IC50 values of 9.10 and 7.50 μM. | 27 |
| Fokienia hodginsii | Twigs and leaves | rare carotane-type sesquiterpenoid, forkienin A (1), new eudesmane-type sesquiterpenoid, forkienin B (2), new natural eudesmane-type sesquiterpenoid, forkienin C | Cytotoxicity against HL-60, SMMC-7721, A-549, MCF-7, and SW480 cell lines. | 28 |
| Fokienia hodginsii | leaves | diterpenoids, trans-communic acid (1), 13-oxo-15,16-dinor-labda-8(17), 11E-diene-19-oic acid (2), 3β-hydroxytotarol (3), and totarolone (4) | Antiproliferative activity in acute myeloid leukemia (OCI-AML) cells | 17 |
| Fokienia hodginsii | | α"Pinene; 1"Octen"3"ol; D"Limonene; α"Terpineol; β"Elemen; Caryophyllene; α"Caryophyllene; β"Cubebene; γ"Cadinene; β"Cadinene; Caryophyllene oxide; τ"Cadinol; α"Cadinol; 7R,8R"8"Hydroxy"4"isopropylidene"7"methylbicyclol | Qi analgesic; Descending retching Epigastric pain, qi vomiting, rheumatoid arthritis, cold headache, abdominal pain. Qi to relieve pain and reduce retinitis | 26,30 |
| Sonchus oleraceus (L.) L and J. sabina L | | | Anti-tumor effects via up-regulation of the apoptosis-related genes FasL, caspase 3 and caspase 9; inhibited expression of ICAM-1, MMP-2 and MMP-9 | 16 |
| Juniperus sabina L. | | Essential oil | Hepatoprotective activity | 17 |

## Slide 2
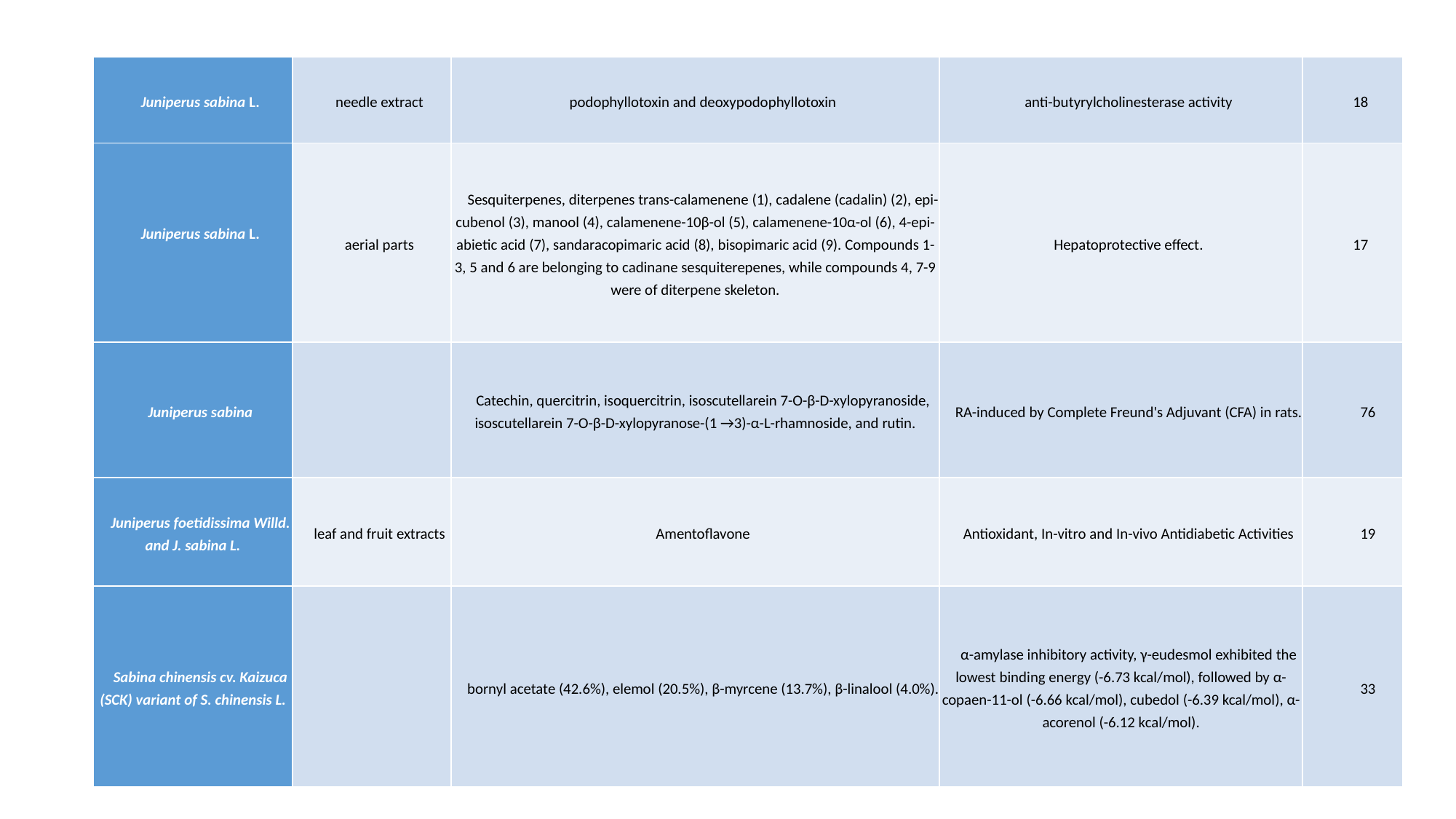

| Juniperus sabina L. | needle extract | podophyllotoxin and deoxypodophyllotoxin | anti-butyrylcholinesterase activity | 18 |
| --- | --- | --- | --- | --- |
| Juniperus sabina L. | aerial parts | Sesquiterpenes, diterpenes trans-calamenene (1), cadalene (cadalin) (2), epi-cubenol (3), manool (4), calamenene-10β-ol (5), calamenene-10α-ol (6), 4-epi-abietic acid (7), sandaracopimaric acid (8), bisopimaric acid (9). Compounds 1-3, 5 and 6 are belonging to cadinane sesquiterepenes, while compounds 4, 7-9 were of diterpene skeleton. | Hepatoprotective effect. | 17 |
| Juniperus sabina | | Catechin, quercitrin, isoquercitrin, isoscutellarein 7-O-β-D-xylopyranoside, isoscutellarein 7-O-β-D-xylopyranose-(1 →3)-α-L-rhamnoside, and rutin. | RA-induced by Complete Freund's Adjuvant (CFA) in rats. | 76 |
| Juniperus foetidissima Willd. and J. sabina L. | leaf and fruit extracts | Amentoflavone | Antioxidant, In-vitro and In-vivo Antidiabetic Activities | 19 |
| Sabina chinensis cv. Kaizuca (SCK) variant of S. chinensis L. | | bornyl acetate (42.6%), elemol (20.5%), β-myrcene (13.7%), β-linalool (4.0%). | α-amylase inhibitory activity, γ-eudesmol exhibited the lowest binding energy (-6.73 kcal/mol), followed by α-copaen-11-ol (-6.66 kcal/mol), cubedol (-6.39 kcal/mol), α-acorenol (-6.12 kcal/mol). | 33 |

## Slide 3
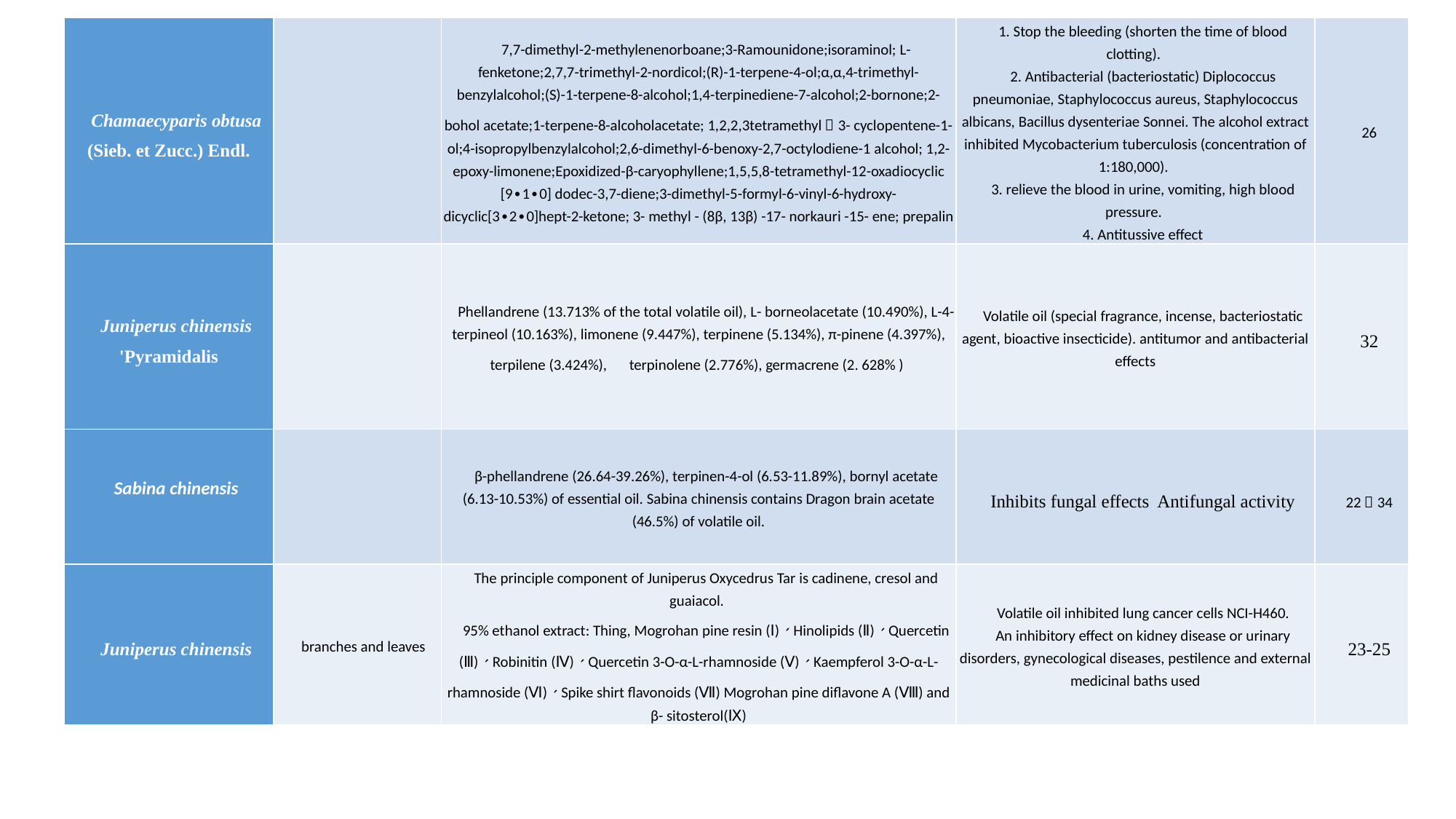

| Chamaecyparis obtusa (Sieb. et Zucc.) Endl. | | 7,7-dimethyl-2-methylenenorboane;3-Ramounidone;isoraminol; L-fenketone;2,7,7-trimethyl-2-nordicol;(R)-1-terpene-4-ol;α,α,4-trimethyl-benzylalcohol;(S)-1-terpene-8-alcohol;1,4-terpinediene-7-alcohol;2-bornone;2-bohol acetate;1-terpene-8-alcoholacetate; 1,2,2,3tetramethyl－3- cyclopentene-1-ol;4-isopropylbenzylalcohol;2,6-dimethyl-6-benoxy-2,7-octylodiene-1 alcohol; 1,2-epoxy-limonene;Epoxidized-β-caryophyllene;1,5,5,8-tetramethyl-12-oxadiocyclic [9∙1∙0] dodec-3,7-diene;3-dimethyl-5-formyl-6-vinyl-6-hydroxy-dicyclic[3∙2∙0]hept-2-ketone; 3- methyl - (8β, 13β) -17- norkauri -15- ene; prepalin | 1. Stop the bleeding (shorten the time of blood clotting). 2. Antibacterial (bacteriostatic) Diplococcus pneumoniae, Staphylococcus aureus, Staphylococcus albicans, Bacillus dysenteriae Sonnei. The alcohol extract inhibited Mycobacterium tuberculosis (concentration of 1:180,000). 3. relieve the blood in urine, vomiting, high blood pressure. 4. Antitussive effect | 26 |
| --- | --- | --- | --- | --- |
| Juniperus chinensis 'Pyramidalis | | Phellandrene (13.713% of the total volatile oil), L- borneolacetate (10.490%), L-4-terpineol (10.163%), limonene (9.447%), terpinene (5.134%), π-pinene (4.397%), terpilene (3.424%),　terpinolene (2.776%), germacrene (2. 628% ) | Volatile oil (special fragrance, incense, bacteriostatic agent, bioactive insecticide). antitumor and antibacterial effects | 32 |
| Sabina chinensis | | β-phellandrene (26.64-39.26%), terpinen-4-ol (6.53-11.89%), bornyl acetate (6.13-10.53%) of essential oil. Sabina chinensis contains Dragon brain acetate (46.5%) of volatile oil. | Inhibits fungal effects Antifungal activity | 22，34 |
| Juniperus chinensis | branches and leaves | The principle component of Juniperus Oxycedrus Tar is cadinene, cresol and guaiacol. 95% ethanol extract: Thing, Mogrohan pine resin (Ⅰ)、Hinolipids (Ⅱ)、Quercetin (Ⅲ)、Robinitin (Ⅳ)、Quercetin 3-O-α-L-rhamnoside (Ⅴ)、Kaempferol 3-O-α-L-rhamnoside (Ⅵ)、Spike shirt flavonoids (Ⅶ) Mogrohan pine diflavone A (Ⅷ) and β- sitosterol(Ⅸ) | Volatile oil inhibited lung cancer cells NCI-H460. An inhibitory effect on kidney disease or urinary disorders, gynecological diseases, pestilence and external medicinal baths used | 23-25 |

## Slide 4
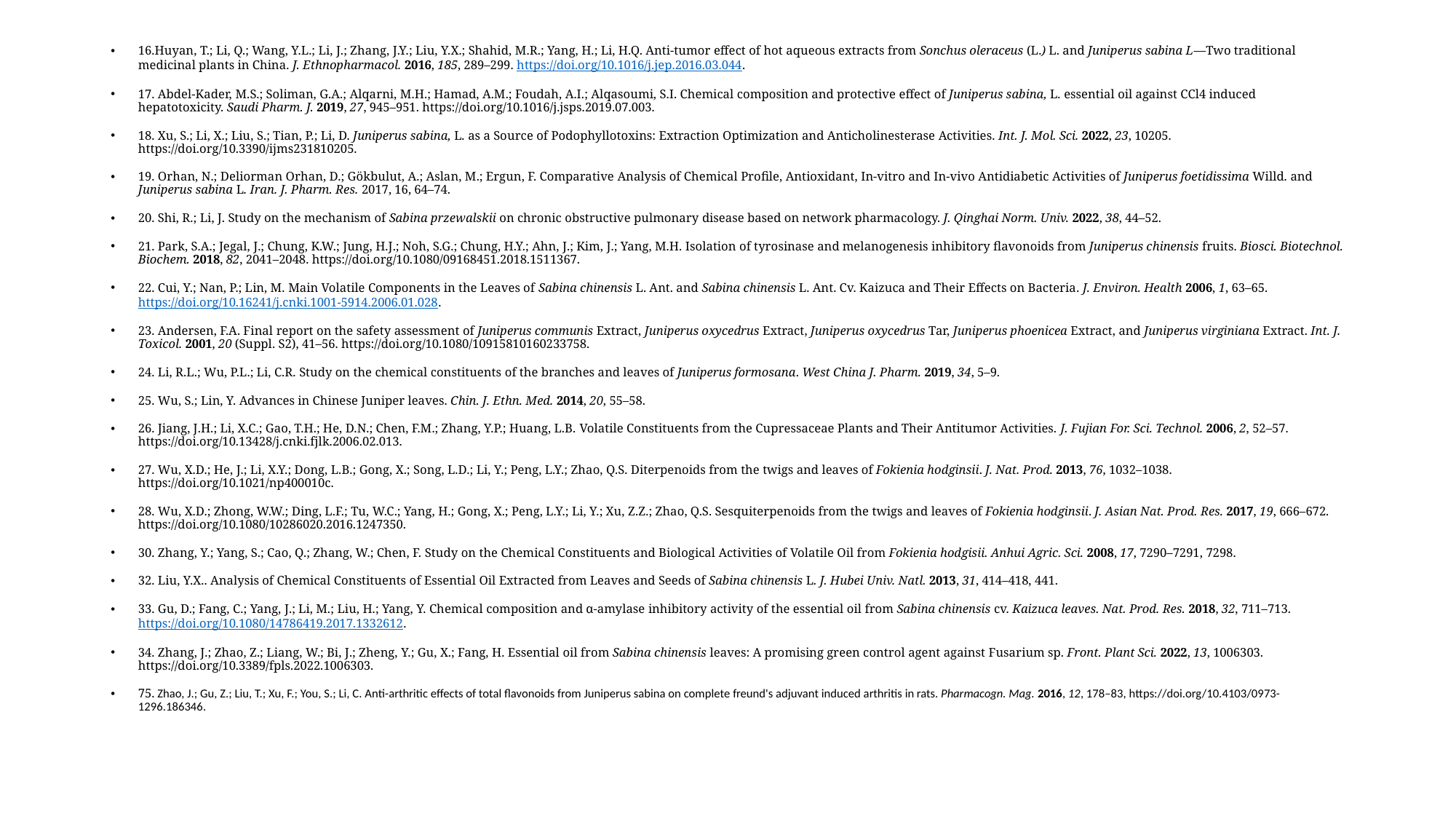

16.Huyan, T.; Li, Q.; Wang, Y.L.; Li, J.; Zhang, J.Y.; Liu, Y.X.; Shahid, M.R.; Yang, H.; Li, H.Q. Anti-tumor effect of hot aqueous extracts from Sonchus oleraceus (L.) L. and Juniperus sabina L—Two traditional medicinal plants in China. J. Ethnopharmacol. 2016, 185, 289–299. https://doi.org/10.1016/j.jep.2016.03.044.
17. Abdel-Kader, M.S.; Soliman, G.A.; Alqarni, M.H.; Hamad, A.M.; Foudah, A.I.; Alqasoumi, S.I. Chemical composition and protective effect of Juniperus sabina, L. essential oil against CCl4 induced hepatotoxicity. Saudi Pharm. J. 2019, 27, 945–951. https://doi.org/10.1016/j.jsps.2019.07.003.
18. Xu, S.; Li, X.; Liu, S.; Tian, P.; Li, D. Juniperus sabina, L. as a Source of Podophyllotoxins: Extraction Optimization and Anticholinesterase Activities. Int. J. Mol. Sci. 2022, 23, 10205. https://doi.org/10.3390/ijms231810205.
19. Orhan, N.; Deliorman Orhan, D.; Gökbulut, A.; Aslan, M.; Ergun, F. Comparative Analysis of Chemical Profile, Antioxidant, In-vitro and In-vivo Antidiabetic Activities of Juniperus foetidissima Willd. and Juniperus sabina L. Iran. J. Pharm. Res. 2017, 16, 64–74.
20. Shi, R.; Li, J. Study on the mechanism of Sabina przewalskii on chronic obstructive pulmonary disease based on network pharmacology. J. Qinghai Norm. Univ. 2022, 38, 44–52.
21. Park, S.A.; Jegal, J.; Chung, K.W.; Jung, H.J.; Noh, S.G.; Chung, H.Y.; Ahn, J.; Kim, J.; Yang, M.H. Isolation of tyrosinase and melanogenesis inhibitory flavonoids from Juniperus chinensis fruits. Biosci. Biotechnol. Biochem. 2018, 82, 2041–2048. https://doi.org/10.1080/09168451.2018.1511367.
22. Cui, Y.; Nan, P.; Lin, M. Main Volatile Components in the Leaves of Sabina chinensis L. Ant. and Sabina chinensis L. Ant. Cv. Kaizuca and Their Effects on Bacteria. J. Environ. Health 2006, 1, 63–65. https://doi.org/10.16241/j.cnki.1001-5914.2006.01.028.
23. Andersen, F.A. Final report on the safety assessment of Juniperus communis Extract, Juniperus oxycedrus Extract, Juniperus oxycedrus Tar, Juniperus phoenicea Extract, and Juniperus virginiana Extract. Int. J. Toxicol. 2001, 20 (Suppl. S2), 41–56. https://doi.org/10.1080/10915810160233758.
24. Li, R.L.; Wu, P.L.; Li, C.R. Study on the chemical constituents of the branches and leaves of Juniperus formosana. West China J. Pharm. 2019, 34, 5–9.
25. Wu, S.; Lin, Y. Advances in Chinese Juniper leaves. Chin. J. Ethn. Med. 2014, 20, 55–58.
26. Jiang, J.H.; Li, X.C.; Gao, T.H.; He, D.N.; Chen, F.M.; Zhang, Y.P.; Huang, L.B. Volatile Constituents from the Cupressaceae Plants and Their Antitumor Activities. J. Fujian For. Sci. Technol. 2006, 2, 52–57. https://doi.org/10.13428/j.cnki.fjlk.2006.02.013.
27. Wu, X.D.; He, J.; Li, X.Y.; Dong, L.B.; Gong, X.; Song, L.D.; Li, Y.; Peng, L.Y.; Zhao, Q.S. Diterpenoids from the twigs and leaves of Fokienia hodginsii. J. Nat. Prod. 2013, 76, 1032–1038. https://doi.org/10.1021/np400010c.
28. Wu, X.D.; Zhong, W.W.; Ding, L.F.; Tu, W.C.; Yang, H.; Gong, X.; Peng, L.Y.; Li, Y.; Xu, Z.Z.; Zhao, Q.S. Sesquiterpenoids from the twigs and leaves of Fokienia hodginsii. J. Asian Nat. Prod. Res. 2017, 19, 666–672. https://doi.org/10.1080/10286020.2016.1247350.
30. Zhang, Y.; Yang, S.; Cao, Q.; Zhang, W.; Chen, F. Study on the Chemical Constituents and Biological Activities of Volatile Oil from Fokienia hodgisii. Anhui Agric. Sci. 2008, 17, 7290–7291, 7298.
32. Liu, Y.X.. Analysis of Chemical Constituents of Essential Oil Extracted from Leaves and Seeds of Sabina chinensis L. J. Hubei Univ. Natl. 2013, 31, 414–418, 441.
33. Gu, D.; Fang, C.; Yang, J.; Li, M.; Liu, H.; Yang, Y. Chemical composition and α-amylase inhibitory activity of the essential oil from Sabina chinensis cv. Kaizuca leaves. Nat. Prod. Res. 2018, 32, 711–713. https://doi.org/10.1080/14786419.2017.1332612.
34. Zhang, J.; Zhao, Z.; Liang, W.; Bi, J.; Zheng, Y.; Gu, X.; Fang, H. Essential oil from Sabina chinensis leaves: A promising green control agent against Fusarium sp. Front. Plant Sci. 2022, 13, 1006303. https://doi.org/10.3389/fpls.2022.1006303.
75. Zhao, J.; Gu, Z.; Liu, T.; Xu, F.; You, S.; Li, C. Anti-arthritic effects of total flavonoids from Juniperus sabina on complete freund's adjuvant induced arthritis in rats. Pharmacogn. Mag. 2016, 12, 178–83, https://doi.org/10.4103/0973-1296.186346.
